# Doubly elastic net regularized online portfolio optimization with transaction costs

**DOI:** 10.1038/s41598-023-46059-2

**Published:** 2023-11-02

**Authors:** Xiaoting Yao, Na Zhang

**Affiliations:** Department of Applied Mathematics College Mathematics and Informatics, South China Agricultural University, Guangzhou, 510642 People’s Republic of China

**Keywords:** Applied mathematics, Computational science, Scientific data, Statistics

## Abstract

Online portfolio optimization with transaction costs is a big challenge in large-scale intelligent computing community, since its undersample from rapidly-changing market and complexity from varying transaction costs. In this paper, we focus on this problem and solve it by machine learning system. Specifically, we reformulate the optimization problem with the minimization over simplex containing three items, which are negative expected return, the elastic net regularization of transaction costs controlled term and portfolio variable, respectively. We propose to apply linearized augmented Lagrangian method (LALM) and the alternating direction method of multipliers (ADMM) to solve the optimization model in a higher efficiency, meanwhile theoretically guarantee their convergence and deduce closed-form solutions of their subproblems in each iteration. Furthermore, we conduct extensive experiments on five benchmark datasets from real market to demonstrate that the proposed algorithms outperform compared state-of-the-art strategies in most cases in six dimensions.

## Introduction

Online portfolio optimization has become a hot topic in the intelligent computing and machine learning community^1–4^. It always obtains high return in the short time with extensive calculation. Hence, there are lots of difficulties such as the undersample in the short time for calculation and the transaction cost problem, which we attack in this paper based on machine learning system.

Portfolio optimization originates from the Markowitz mean-variance (MV) theory^5^. However, the online portfolio problem can not be managed by most extended MV theories, since its instantaneous transaction data can not provide a stable and reliable distribution to estimate significant financial parameters. Besides, it is difficult to guarantee the generalization of the optimization model, since online portfolio selection problem is in the rapidly-changing circumstance. In this case, we usually have only a small window to make decision and always undersample for training. Nevertheless, online portfolio optimization is attractive and more general in reality, since its timely and sensitive response to the dynamic market. One common way to solve above problems is decomposing raw data structure to extract the return or risk information. Chen et al.^6^ exploited wavelet feature engineering to transform stock price data, which resulted in higher Sharpe ratio in long-short portfolio trading. Lai et al.^7^ extracted instantaneous risk structure from price covariance matrix, to improve profit by lost control. Another common way to manage online portfolio problem is using machine learning methods. Kelly’s criterion in the information theory^8–10^ brings out the exponential growth rate (EGR) approach, which focuses on the wealth change integrated with online learning frameworks^1,11,12^. The EGR approach is suitable for online portfolio optimization for the reason that it always works via data-driven rather than requiring strict statistical assumptions. Recently, researchers focus more on various machine learning schemes^13–16^ in the basis of EGR approach. Zou et al.^17^ proposed that elastic net (the linear combination of L1 norm and the square of L2 norm) leads to sparsity while maintaining a higher accuracy. Ho et al.^18^ applied elastic net for portfolio optimization, and Shen et al.^19^ constructed sparse portfolio with doubly norm regularized terms, showing an excellent performance. Hence, elastic net interests us and we illustrate it in the following text. Furthermore, researches^20,21^ indicate that regularized the portfolio variable contributes to the generalization of the optimization model, which we would also elaborate in the following. Lai et al.^22^ applied L1 regularized on the portfolio variable and resulted sparse and stable portfolios in the case of short-term investment. Moreover, Luo et al.^23^ regularized portfolio variable by L0 norm while showing higher sparsity and better return in online portfolio selection.

However, above strategies even most portfolio strategies do not consider transaction costs, which could not be ignored (especially in online portfolio problem) in reality. It is worth noting that transaction costs can be classified into explicit and implicit. Implicit transaction costs are usually caused by market impact or liquidity risk^24,25^, and the explicit is from the transaction. Particularly, we only focus on explicit transaction costs, such as taxes^26^, buying and selling transaction fees, since they influence more on retailers while we incline to research the online portfolio for retailers.

The first explicit extension was proposed by Bauer et al.^27^ that Cover’s Universal Portfolio^9^ is still suitable for imposing transaction costs. However, they did not take transaction costs into the decision process. Albeverio et al.^28^ proposed a new transaction costs optimization model, where reformulated transaction costs as the distance between portfolios. Certainly, it is widely to manage transaction costs via machine learning system. Györfi et al.^29^ augmented the original Markowitz objective function by adding a penalty term proportional to the sum of the absolute of the portfolio weights, which encourage sparse portfolios and allow transaction costs to be considered. Das et al.^30^ applied machine learning method to study transaction costs problem, and solve it by GP algorithm. Furtherly, Li et al.^31^ solve portfolio selection problem with transaction costs by proposing a TCO framework, which can get closed-form formulae for portfolio update, and they also found the relationship between transaction costs and portfolios.

Although online portfolio optimization have been studied for decades, it lacks of models considering both transaction costs and sparsity as well as correlations of portfolio variables, simultaneously. Thus, we aim to design in this paper a mathematical model concerning transaction costs as well as regularization of portfolios to enhance the cumulative net wealth and the generalization ability of the model. Noting that elastic net regularization can perform automatic variable selection and maintain related variable groups, we propose a doubly elastic net regularized model for portfolio selection problems. We then apply the linearized augmented Lagrangian method (LALM) and alternating direction method of multipliers (ADMM) to solve the proposed model. It is worth mentioning that guaranteeing the solving algorithm converging to the corresponding model is difficult. A number of researches^18,19,31^ did not prove the convergence of the algorithm theoretically, but we do that. Numerical experiments show the efficiency of the proposed algorithms. The innovations and main contributions of this paper are as follows:

- We propose a minimization problem over simplex which concerns transaction costs and regularization of portfolios simultaneously. The objective of the problems contains three terms: the negative expected return, the elastic net regularization of the difference between the portfolios of the next and last periods to control the transaction cost, the elastic net regularization (reduces to the square of L2 norm due to the simplex) of the portfolio vector to improve the generalization of the model. Thanks to the properties of the elastic net, the proposed model considers the transaction cost, the sparse property and the correlation between variables simultaneously.
- In order to solve the proposed model, we apply the LALM to the model and demonstrate the sequence generated by the algorithm converges to a solution of the proposed model. Further, the closed-form solution of the subproblem in each iteration is established, enabling the computational efficiency of the algorithm. We point out that LALM does not need to project any vector to the simplex set in each iteration, which may save computational time in practice.
- We further apply ADMM to solve the proposed model by appropriately splitting the variable into two variables. The convergence of ADMM is established through proving the existence of saddle point of the corresponding augmented Lagrangian function. Different from LALM, each update of ADMM is restricted to the simplex set, which may improve the accuracy of the algorithm.
- We compare the proposed algorithms with the state-of-the-art methods for portfolio selection on four benchmark datasets. Numerical experiments illustrate that the proposed algorithms perform better than other compared methods in most cases.

This paper is organized as follows. We present some preliminaries and related works in section “Preliminaries”. The whole portfolio selection system and its solving algorithms are illustrated in section “Portfolio optimization”. Section “Experiment” focuses on the experimental results to evaluate the efficiency of our proposed algorithms and section “Conclusion” summarizes the paper.

## Preliminaries

### Problem setting

In the real market, transaction costs can not be ignored especially in the short-term investment. Assuming that there are *m* assets invested for *T* periods in a financial market. The relative prices can be collected as a vector $\mathbf {x_t}=(x_{t,1},\ldots ,x_{t,i},\ldots ,x_{t,m}),\ t=1,2,\cdots ,T$, where $x_{t,i}=\frac{P_{t,i}}{P_{t-1,i}}$ denotes the relative price and $P_{t,i}$ is the closed price of *i* *th* asset in *t* *th* period, respectively. A portfolio vector $\mathbf {b_t}=(x_{b,1},\ldots ,x_{b,i},\ldots ,x_{b,m})$ is restricted in the simplex $\bigtriangleup _m=\{{\textbf{b}}: {\textbf{b}}\in {\mathbb {R}}_+^m,\ \sum _{i=1}^{m}b_i=1 \}$ with assumptions of self-financed and non-margin and non-shorting, where $b_{t,i}$ indicates the proportion of total wealth invested in *i* *th* asset of *t* *th* period.

At the beginning of *t* *th* period, the portfolio is set to $\mathbf {b_t}$, thus the wealth allocation at the end is changed to ${\hat{b}}_{t}=\frac{\mathbf {b_t}\centerdot \mathbf {x_t}}{<\mathbf {b_t},\mathbf {x_t}>}$, where $<\cdot \ ,\ \cdot>$ is the inner product and $\cdot$ is the element-wise product, since the fluctuation of market without rebalancing the portfolio during $t^{th}$ period. Supposing transaction costs denoted as $\gamma$, researches^27–29,31^ proposed the relationship of $\gamma$ and wealth:

1 $$\begin{aligned} 1=w_{t-1}+\gamma \vert \vert {\hat{\textbf{b}}_{t-1}}-\mathbf {b_t} w_{t-1}\vert \vert _1\, \end{aligned}$$

where $w_{t-1}$ denotes the net proportion wealth after transaction costs, and $\vert \vert \cdot \vert \vert _p$ denotes the ${\mathcal {l}}_p$ norm. Furthermore, Li et al.^31^ pointed out that final cumulative wealth implicated transaction costs should be updated as:

2 $$\begin{aligned} S_T=S_0\prod _{t=1}^{T}\left( <\mathbf {b_t}, \mathbf {x_t}>w_{t-1}\right) \, \end{aligned}$$

where $S_0$ is always normalized to 1. $S_T$ is also called net cumulative wealth, which possesses more computing and comparing significance.

### Related works

#### Brenchmarks systems

Uniformly buy-and-hold (UBAH), Beststock (BEST) and Best Constant Rebalanced Portfolios (BCRP) are three benchmark portfolio strategies. UBAH is a simple but widely-used system, which invests evenly at the beginning and holds until the whole period: ${\textbf{b}}_1=\left( \frac{1}{m},\ldots ,\frac{1}{m}\right)$. Thus, it is obviously that the cumulative wealth of UBAH is $S_T={\textbf{b}}_1 \left( \odot _{i=1}^T{\textbf{x}}_t \right)$, where $\odot$ denotes the element-wise product. Similarly but differently, BEST invests totally in the best outperformance asset in hindsight and remains unchanged: ${\textbf{b}}_1=\mathop {\arg \max }\limits _{\mathbf {b \in \bigtriangleup _m}}\ {\textbf{b}} \left( \odot _{i=1}^T{\textbf{x}}_t \right)$ and $S_T=\mathop {\max }\limits _{\mathbf {b \in \bigtriangleup _m}}\ {\textbf{b}} \left( \odot _{i=1}^T{\textbf{x}}_t \right)$. Besides, BCRP updates portfolio to ${\textbf{b}}^*=\mathop {\arg \max }\limits _{\mathbf {b \in \bigtriangleup _m}}\ \sum _{t=1}^{T}\log ({\textbf{b}}^T{\textbf{x}}_t)$ in each period, where ${\textbf{b}}^*$ denotes the portfolio maximizing return in hindsight. Therefore, the cumulative wealth of BCRP is $S_T=\prod _{t=1}^{T}\left( {\textbf{b}}^{*T}{\textbf{x}}_t \right)$.

#### Related works on transaction costs

Gy$\mathrm {\ddot{o}}$rfi et al.^29^ extended the Markowitz portfolio framework by adding penalty term to allow proportional transaction costs considered. The model is widely adopted and attracts an amount of attraction in the area of controlling transaction costs by using penalty term in the portfolio selection problem. They denoted transaction costs related factor as the ratio of net wealth after rebalancing to wealth before rebalancing, that is, $w_{t-1}=\frac{N_{t-1}}{S_{t-1}}$. The rate of proportional transaction costs during sales and buys were denoted by $0<c_s<1$ and $0<c_b<1$, which are controlled by the following formula

3 $$\begin{aligned} 1=w_{t-1}+c_s\sum _{i=1}^{m}\left( \frac{b_{t-1,i}x_{t-1,i}}{{\textbf{b}}_{t-1}\cdot {\textbf{x}}_{t-1}}-b_{t,i}w_{t-1}\right) ^+ +c_b\sum _{i=1}^{m}\left( b_{t,i}w_{t-1}-\frac{b_{t-1,i}x_{t-1,i}}{{\textbf{b}}_{t-1}\cdot {\textbf{x}}_{t-1}}\right) ^+. \end{aligned}$$

They proposed the recursive portfolio strategy as follows

4 $$\begin{aligned} {\textbf{b}}_{t+1}=\mathop {\arg \max }\limits _{\mathbf {b'}}\ v({\textbf{b}}_{t},{\textbf{b}}',{\textbf{X}}_t)+(1-\delta _t)\textrm{E}\{F_{\delta _t}({\textbf{b}}',{\textbf{X}}_{t+1})\vert {\textbf{X}}_t\}, \end{aligned}$$

where $F_{\delta }({\textbf{b}},{\textbf{X}})$ is the recursive function formulated by the discounted Bellman equation:

5 $$\begin{aligned} F_{\delta }({\textbf{b}},{\textbf{X}})=\max _{\mathbf {b'}}\ v({\textbf{b}},{\textbf{b}}',{\textbf{x}})+(1-\delta )\textrm{E}\{F_{\delta }({\textbf{b}}',{\textbf{X}}_{2})\vert {\textbf{X}}_1={\textbf{x}}\}, \end{aligned}$$

where $\delta _t$ is a discount factor such that $\delta _t \rightarrow 0$, ${{\textbf{X}}_t}$ is the homogeneous and first order Markov process, and $v({\textbf{b}},{\textbf{b}}',{\textbf{x}})$ is the inner function defined in^29^.

Li et al.^31^ considered another proportional transaction cost model named Transaction Cost Optimization (TCO), which is a sparse portfolio selection model by adding norm penalized. They took transaction costs as the regularization term of the portfolio model by L1 norm penalized, and obtained the closed-form solution of portfolio update through proximal gradient descent method. Li et al. theoretically guaranteed that transaction cost is related to $\vert \vert {\textbf{b}}-{\hat{\textbf{b}}}_t\vert \vert _1$ by proving

6 $$\begin{aligned} \begin{aligned} \frac{1-\gamma }{1-\gamma +\gamma \vert \vert {\hat{\textbf{b}}_{t-1}}-\mathbf {b_t}\vert \vert _1}\le w_{t-1} \le \frac{1+\gamma }{1+\gamma +\gamma \vert \vert {\hat{\textbf{b}}_{t-1}}-\mathbf {b_t}\vert \vert _1}\, \end{aligned} \end{aligned}$$

for which indicated that the net proportion is inversely related to $\vert \vert {\textbf{b}}-{\hat{\textbf{b}}}_t\vert \vert _1$. Thus they proposed the following model:

7 $$\begin{aligned}{} & {} {\textbf{b}}_{t+1}=\mathop {\arg \min }\limits _{{\textbf{b}}}\ -{\textrm{E}}\{\log {\textbf{b}}\cdot \tilde{{\textbf{x}}}_{t+1}\}+\lambda \vert \vert {\hat{\textbf{b}}_{t-1}}-\mathbf {b_t}\vert \vert _1 \nonumber \\{} & {} s.t.\ {\textbf{b}}\cdot {\textbf{1}}=1,\ {\textbf{b}}\ge 0\, \end{aligned}$$

where $\tilde{{\textbf{x}}}_{t+1}$ denotes the predicted price relative vector and $\lambda$ is a regularization parameter. They acquired the closed-form solution in the following:

8 $$\begin{aligned} \tilde{{\textbf{b}}}_{t+\frac{1}{2}}&=\eta _t\left( {\textrm{E}}\left\{ \frac{\tilde{{\textbf{x}}}_{t+1}}{{\hat{\textbf{b}}}_t\cdot \tilde{{\textbf{x}}}_{t+1}}\right\} -\frac{1}{m}{\textbf{1}}\cdot {\textrm{E}}\left\{ \frac{\tilde{{\textbf{x}}}_{t+1}}{{\hat{\textbf{b}}}_t\cdot \tilde{{\textbf{x}}}_{t+1}}\right\} \right) , \end{aligned}$$

9 $$\begin{aligned} {\textbf{b}}_{t+1}&={\hat{\textbf{b}}}_t+sign\left( \tilde{{\textbf{b}}}_{t+\frac{1}{2}}\right) \left[ \vert \tilde{{\textbf{b}}}_{t+\frac{1}{2}}\vert -\lambda \eta _{t+\frac{1}{2}}\right] _+, \end{aligned}$$

where $\eta _t$ is an inner variable, $[v]_+=\max (0,v)$, and *sign*(*v*) denotes the sign of *v*.

## Portfolio optimization

### Price information

We should consider price forecasting method to achieve the data-driven ideology, which can lessen the influence by irrational factors^32–34^ in the market. Specifically, we formulate the expected return by price forecasting method based on historical information.

PAMR^35^ and CWMR^12^ showed that the predicted relative price in next period is inversely to the current period: $\mathbf {x_{t+1}}=\frac{1}{\mathbf {x_t}}$, which utilized the properties of single-period mean reversion to balance risk and return. Besides, OLMAR^3^ exploited muti-period mean reversion to solve the moving case. It proposed that the relative price in next period will revert to the moving average:

10 $$\begin{aligned} \mathbf {x_{t+1}}=\frac{1}{w}\left( {\textbf{1}}+\frac{1}{\mathbf {x_t}}+\cdots +\frac{1}{\odot _{i=0}^{w-2}\mathbf {x_{t-i}}}\right) , \end{aligned}$$

where *w* is the window size, and it smooths the price volatility in online portfolio problem.

Moreover, above mean reversion strategies may be sub-optimal subject to the noise from real market, due to the real market is not normally distributed^36^. Meanwhile, the robust median reversion (RMR)^37^ is robust to real market and can withstand nontrivial transaction costs, which utilizes L1 median estimator^38,39^ and online machine learning. RMR can solve the long tail distribution of real market and is shown below:

11 $$\begin{aligned} \mathbf {x_{t+1}}=\left( \mathop {\arg \min }\limits _{\textbf{p}} \sum _{i=0}^{w-1}\vert \vert {\textbf{P}}_{t-i}-{\textbf{P}}\vert \vert _2\right) /\mathbf {x_{t}}. \end{aligned}$$

On the other hand, most investors will follow the trend and keep purchasing rising stocks, thus they always consider ${\textbf{P}}_{MAX}$ as a potential level that the future price can probably reach. Consequently, a generalized logarithmic return (GLR)^22^ was proposed to predict the relative price in next period:

12 $$\begin{aligned} \mathbf {x_{t+1}}=1.1\log \left( \frac{{\textbf{P}}_{MAX}}{{\textbf{P}}_t}\right) +1. \end{aligned}$$

In order to illustrate the adaptability to various price processes of the optimization model, and comprehensively predict the relative price, we adopt in the paper above methods to further consider the online portfolio optimization with transaction costs.

### The proposed doubly elastic net regularized online portfolio optimization with transaction costs

Considering the goals of maximizing cumulative wealth and minimizing transaction costs, the portfolio model we build in this paper as follows

13 $$\begin{aligned} \begin{aligned} {\textbf{b}}_{t+1}\in \ \mathop {\arg \min }\limits _{\mathbf {b \in \bigtriangleup _m}} \ -{\textbf{f}}^T{\textbf{b}}+\lambda \vert \vert {\textbf{b}}-{\hat{\textbf{b}}_{t}}\vert \vert _1+\frac{\eta }{2}\vert \vert {\textbf{b}}-{\hat{\textbf{b}}_{t}}\vert \vert _2^2+\frac{\tau }{2}\vert \vert {\textbf{b}}\vert \vert _2^2\, \end{aligned} \end{aligned}$$

where $\lambda >0$, ${\textbf{f}}$ is the predicted relative price in the next period. Here, we consider the following four cases: ${\textbf{f}}=\frac{1}{\mathbf {x_t}}$, ${\textbf{f}}=\frac{1}{w}\left( 1+\frac{1}{\mathbf {x_t}}+\cdots +\frac{1}{\odot _{i=0}^{w-2} \mathbf {x_{t-i}}}\right)$, ${\textbf{f}}=(\mathop {\arg \min }\limits _{\textbf{p}} \sum _{i=0}^{w-1}\vert \vert {\textbf{P}}_{t-i}-{\textbf{P}}\vert \vert _2)/\mathbf {x_{t}}$ and ${\textbf{f}}=1.1\log \left( \frac{{\textbf{P}}_{MAX}}{{\textbf{P}}_t}\right) +1$. Since model (13) involves in the elastic net regularization terms for ${\textbf{b}}-{\hat{\textbf{b}}_{t}}$ and ${\textbf{b}}$, which will be explained below, we call model (13) the doubly elastic net regularized portfolio optimization (DENRPO) model.

It can be found that ${\textbf{f}}^T{\textbf{b}}$ represents the predicted wealth increasing factor, implying expected return potential of the whole portfolio. Therefore, the goal of maximizing cumulative wealth can be reformulated to this item and negative expected return can change the maximization to a minimization.

Further, we implement the proposed model to manage transaction costs with better generalization. On one hand, transaction costs can be reflected by the wealth growth denoted by net asset proportion. Inspired by the research in that transaction costs influent the net proportion by the distance of $\mathbf {b_t}$ and ${\hat{\textbf{b}}_{t-1}}$, we innovatively apply elastic net for this term, since it makes a difference in the following aspect. Firstly, elastic net tends to preserve the highly correlated variables by L2 norm structure while maintaining sparsity by L1 norm, thus it can avoid extreme positions and improve the diversification and stability. Besides, it is according to regression analysis that elastic net is particularly useful when assets are large-scale, since it overcomes poor sample sentiments. The above analyses lead to minimize

$$\begin{aligned} \lambda \vert \vert {\textbf{b}}-{\hat{\textbf{b}}_{t}}\vert \vert _1+\frac{\eta }{2}\vert \vert {\textbf{b}}-{\hat{\textbf{b}}_{t}}\vert \vert _2^2 \end{aligned}$$

to trade off transaction costs, in which $\lambda$ and $\eta$ are the regularization parameters controlling transaction costs. The smaller the $\lambda$ and $\eta$ are will the smaller the regularization strength is, meanwhile indicating that the model is more inclined to obtain high returns. When the $\lambda$ and $\eta$ are larger, these regularization terms will make $\mathbf {b_{t+1}}$ more inclined to ${\hat{\textbf{b}}_{t}}$, that is the number of rebalanced assets is reduced, thus lessens the transaction cost.

On the other hand, researches show that the portfolio variable regularization $\vert \vert {\textbf{b}}\vert \vert$ makes sense in the portfolio optimization. Fan et al.^40^ showed that L1 norm penalty on portfolio variable is equivalent to constraining the risk or utility cumulative statistical estimation error, since constraints on individual assets limits total exposure, thus controlling for risk approximation errors as well as closer the empirical and actual risk. In addition, Brodie et al.^20^ proposed that portfolio weights can represent transaction costs. Furthermore, Li^41^ proposed that penalizing portfolio weights favors the sparsity and stability of portfolio, since shifting and scaling the portfolio weights derived from the sample estimates towards zero allows small portfolio weights to be set to zero and extremely large positions to be regulated, resulting in sparse and stable portfolios. Besides, DeMiguel et al.^42^ showed that the global minimum variance of a portfolio can be generated through the portfolio regularization term. The above researches provide us with ideas for constructing the model, since this regularization contributes to the generalization of the portfolio model can be theoretically guaranteed. Thus we apply elastic net for ${\textbf{b}}$, which the form is the combination of L1 norm and the square of L2 norm for the portfolio vector with some tuning parameters, as the regularization term of the proposed model. Since ${\textbf{b}}$ is restricted to the simplex, the first term of the elastic net is equivalent to the constant 1. Therefore, we need to minimize $\frac{\tau }{2}\vert \vert {\textbf{b}}\vert \vert _2^2$ to control the sparsity and stability of our model, in which $\tau$ is the regularization parameter controlling the generalization of the model. Based on above discussions, we propose optimization model (13).

The next theorem establishes the existence of solutions to the problem (13).

#### Theorem 1

For $\lambda ,\ \eta ,\ \tau \ge 0$, problem (13) has optimal solutions. Further, if $\eta >0$ or $\tau >0$, problem (13) has a unique optimal solution.

#### Proof

It is easy to know that the objective function in model (13) is continuous and the constrain $\bigtriangleup _m$ is closed and bounded. Thus we can obtain that problem (13) has optimal solutions. If $\eta >0$ or $\tau >0$, the objective function is strongly convex. Therefore, problem (13) has a unique optimal solution. $\square$

### Solving algorithms

In this section, we develop algorithms to solve the problem (13) in a higher efficiency.

#### Linearized augmented Lagrangian method

Augmented Lagrangian method (ALM) is an excellent algorithm due to its efficiency for solving the linear equality constrained optimization problem. Hence, we apply the ALM to solve the proposed model (13). Since the nonlinear term in the model will increase the solving difficulty of using general ALM, we develop a linearized augmented Lagrangian method (LALM), which linearizes the quadratic term of ALM, to solve the problem in a higher efficiency.

We first introduce the notion of indicator function on ${\mathbb {R}}_+^m$, denoted by ${\textbf{I}}_{{\mathbb {R}}_+^m}$, which is defined at ${\textbf{b}} \in {\mathbb {R}}^m$ by

14 $$\begin{aligned} {\textbf{I}}_{{\mathbb {R}}_+^m}({\textbf{b}})= {\left\{ \begin{array}{ll} 0, \hspace{5mm}&{}{\textbf{b}} \in {\mathbb {R}}_+^m,\\ +\infty , &{} \textrm{otherwise}. \end{array}\right. } \end{aligned}$$

By this way, the augmented Lagrangian function of problem (13) is

15 $$\begin{aligned} \begin{aligned} L({\textbf{b}},\xi )=&-{\textbf{f}}^T{\textbf{b}}+\lambda \vert \vert {\textbf{b}}-{\hat{\textbf{b}}_{t}}\vert \vert _1+\frac{\eta }{2}\vert \vert {\textbf{b}}-{\hat{\textbf{b}}_{t}}\vert \vert _2^2+\frac{\tau }{2}\vert \vert {\textbf{b}}\vert \vert _2^2+{\textbf{I}}_{{\mathbb {R}}_+^m}({\textbf{b}})+\xi ({\textbf{1}}^T{\textbf{b}}-1) +\frac{\rho }{2} ({\textbf{1}}^T {\textbf{b}}-1)^2\, \end{aligned} \end{aligned}$$

where $\xi \in {\mathbb {R}}$ is the Lagrange multiplier and $\rho >0$ is a penalty parameter. Then, the LALM updates ${\textbf{b}}^{k+1}$ and $\xi ^{k+1}$ in each iteration by

16 $$\begin{aligned}{} & {} {\textbf{b}}^{k + 1} \in \mathop {\arg \min }\limits _{{\textbf{b}}}\ g({\textbf{b}})+\xi ^{k}({\textbf{1}}^{T}{\textbf{b}}- 1)+< {\textbf{b}} - {\textbf{b}}^{k}, \rho {\textbf{1}} ({\textbf{1}}^{T}{\textbf{b}}^{k}-1)>+\frac{1}{2\alpha }\vert \vert {\textbf{b}} - {\textbf{b}}^{k}\vert \vert _{2}^{2}\, \end{aligned}$$

17 $$\begin{aligned}{} & {} \xi ^{k+1}= \xi ^k+\rho ({\textbf{1}}^T{\textbf{b}}^{k+1}-1)\, \end{aligned}$$

where $g({\textbf{b}})=-{\textbf{f}}^T{\textbf{b}}+\lambda \vert \vert {\textbf{b}}-{\hat{\textbf{b}}_{t}}\vert \vert _1+\frac{\eta }{2}\vert \vert {\textbf{b}}-{\hat{\textbf{b}}_{t}}\vert \vert _2^2+\frac{\tau }{2}\vert \vert {\textbf{b}}\vert \vert _2^2+{\textbf{I}}_{{\mathbb {R}}_+^m}({\textbf{b}})$, and $\alpha$ is a parameter which will be described in the following text.

The following lemma tells us that the problem (16) can be computed in an easy way. Specifically, the closed-form solution of problem (16) only involves in the soft-thresholding operator and the projection onto ${\mathbb {R}}_+^m$.

##### Lemma 1

The closed-form solution for the optimization problem (16) is

18 $$\begin{aligned} {\textbf{b}}^{k+1}={\textrm{P}}_{{\mathbb {R}}_+^m}\left\{ \ {\hat{\textbf{b}}_{t}}+\textrm{sign}({\textbf{q}})\left[ \vert {\textbf{q}}\vert -\frac{\lambda }{\tau +\eta +\frac{1}{\alpha }}\right] _+\right\} \, \end{aligned}$$

where $([{\textbf{x}}]_+)_i=max\{{\textbf{x}}_i,0\}$ for $i=1,\ldots ,m$ and ${\textbf{x}}\in {\mathbb {R}}_+^m$,

$$\begin{aligned} \begin{aligned} {\textbf{q}}=&\frac{\eta }{\tau +\eta +\frac{1}{\alpha }}{\hat{\textbf{b}}_t}+\frac{\frac{1}{\alpha }}{\tau +\eta +\frac{1}{\alpha }}{\textbf{b}}^k-\frac{\rho }{\tau +\eta +\frac{1}{\alpha }}{\textbf{1}}({\textbf{1}}^T{\textbf{b}}^k-1)+\frac{1}{\tau +\eta +\frac{1}{\alpha }}{\textbf{f}}-\frac{1}{\tau +\eta +\frac{1}{\alpha }}{\textbf{1}}\xi ^k-{\hat{\textbf{b}}_{t}} \, \end{aligned} \end{aligned}$$

and $\textrm{P}_{{\mathbb {R}}_+^m}$ is the projection onto ${\mathbb {R}}_+^m$.

##### Proof

We derive from (16) that

19 $$\begin{aligned} \begin{aligned} {\textbf{b}}^{k+1}\in&\mathop {\arg \min }\limits _{{\textbf{b}}\in {\mathbb {R}}_+^m} \lambda \vert \vert \textbf{b}-\hat{\textbf{b}_\textbf{t}}\vert \vert _1+\frac{\eta }{2}\vert \vert \textbf{b}-\hat{\textbf{b}_\textbf{t}}\vert \vert _2^2+\frac{\tau }{2}\vert \vert {\textbf{b}}\vert \vert _2^2+\frac{1}{2\alpha }\vert \vert {\textbf{b}}-{\textbf{b}}^k\vert \vert _2^2+<{\textbf{b}},\rho {\textbf{1}}({\textbf{1}}^{T}{\textbf{b}}^{k}-1)-{\textbf{f}}+{\textbf{1}}\xi ^k> \\ =\,&\mathop {\arg \min }\limits _{{\textbf{b}}\in {\mathbb {R}}_+^m} \lambda \vert \vert \textbf{b}-\hat{\textbf{b}_\textbf{t}}\vert \vert _1+\frac{\eta }{2}\vert \vert \textbf{b}-\hat{\textbf{b}_\textbf{t}}\vert \vert _2^2+\frac{\tau }{2}\vert \vert {\textbf{b}}\vert \vert _2^2+\frac{1}{2\alpha }\vert \vert {\textbf{b}}-{\textbf{b}}^k\vert \vert _2^2+<{\textbf{b}},{\textbf{d}}> \, \end{aligned} \end{aligned}$$

where ${\textbf{d}}=\rho {\textbf{1}}({\textbf{1}}^{T}{\textbf{b}}^{k}-1)-{\textbf{f}}+{\textbf{1}}\xi ^k$. Then the formula (19) can be further changed into

20 $$\begin{aligned} \begin{aligned} {\textbf{b}}^{k+1}\in&\mathop {\arg \min }\limits _{{\textbf{b}}\in {\mathbb {R}}_+^m} \lambda \vert \vert \textbf{b}-\hat{\textbf{b}_\textbf{t}}\vert \vert _1+\frac{1}{2}\left( \tau +\eta +\frac{1}{\alpha }\right) \vert \vert {\textbf{b}}-\frac{\eta {\hat{\textbf{b}}_t}}{\tau +\eta +\frac{1}{\alpha }}-\frac{\frac{1}{\alpha }{\textbf{b}}^k}{\tau +\eta +\frac{1}{\alpha }}+\frac{{\textbf{d}}}{\tau +\eta +\frac{1}{\alpha }}\vert \vert _2^2\\ =&\mathop {\arg \min }\limits _{{\textbf{b}}\in {\mathbb {R}}_+^m} \lambda \vert \vert \textbf{b}-\hat{\textbf{b}_\textbf{t}}\vert \vert _1+\frac{1}{2}\left( \tau +\eta +\frac{1}{\alpha }\right) \vert \vert {\textbf{b}}-{\textbf{w}}\vert \vert _2^2\, \end{aligned} \end{aligned}$$

where ${\textbf{w}}=\frac{\eta }{\tau +\eta +\frac{1}{\alpha }}{\hat{\textbf{b}}_t}+\frac{\frac{1}{\alpha }}{\tau +\eta +\frac{1}{\alpha }}{\textbf{b}}^k-\frac{1}{\tau +\eta +\frac{1}{\alpha }}{\textbf{d}}$. Afterwards, let ${\textbf{u}}=\textbf{b}-\hat{\textbf{b}_\textbf{t}}$, then we can find that

21 $$\begin{aligned} \begin{aligned} {\textbf{u}}^{k+1}\in&\mathop {\arg \min }\limits _{{\textbf{u}}\in \textrm{T}} \lambda \vert \vert {\textbf{u}}\vert \vert _1+\frac{1}{2}\left( \tau +\eta +\frac{1}{\alpha }\right) \vert \vert {\textbf{u}}-({\textbf{w}}-\hat{\textbf{b}_\textbf{t}})\vert \vert _2^2\\ =\,&\mathop {\arg \min }\limits _{{\textbf{u}}\in \textrm{T}}\frac{\lambda }{\tau +\eta +\frac{1}{\alpha }}\vert \vert {\textbf{u}}\vert \vert _1+\frac{1}{2}\vert \vert {\textbf{u}}-({\textbf{w}}-\hat{\textbf{b}_\textbf{t}})\vert \vert _2^2\\ =\,&\mathop {\arg \min }\limits _{{\textbf{u}}\in \textrm{T}}\frac{\lambda }{\tau +\eta +\frac{1}{\alpha }}\vert \vert {\textbf{u}}\vert \vert _1+\frac{1}{2}\vert \vert {\textbf{u}}-{\textbf{q}}\vert \vert _2^2\\ =\,&\textrm{P}_{\textrm{T}}\left\{ \ \textrm{sign}({\textbf{q}})\left[ \vert {\textbf{q}}\vert -\frac{\lambda }{\tau +\eta +\frac{1}{\alpha }}\right] _+\right\} \, \end{aligned} \end{aligned}$$

where ${\rm P}_{\rm T}$ is the projection onto the set $\textrm{T}$, ${\textbf{q}}={\textbf{w}}-\hat{\textbf{b}_\textbf{t}}$, that is

$$\begin{aligned} {\textbf{q}}=&\frac{\eta }{\tau +\eta +\frac{1}{\alpha }}{\hat{\textbf{b}}_t}-{\hat{\textbf{b}}_t}+\frac{\frac{1}{\alpha }}{\tau +\eta +\frac{1}{\alpha }}{\textbf{b}}^{k}-\frac{1}{\tau +\eta +\frac{1}{\alpha }}{\textbf{1}}\xi ^{k}-\frac{\rho }{\tau +\eta +\frac{1}{\alpha }}{\textbf{1}}({\textbf{1}}^T{\textbf{b}}^{k}-1)+\frac{1}{\tau +\eta +\frac{1}{\alpha }}{\textbf{f}}\ , \end{aligned}$$

and $$\textrm{T}=\{{\textbf{u}}\in {\mathbb {R}}^n:{\textbf{u}}+{\hat{\textbf{b}}}_t \in {\mathbb {R}}^m_+\}$$.

Then

$$\begin{aligned} \begin{aligned} {\textbf{b}}^{k+1}=&{\hat{\textbf{b}}}_t+{\textbf{u}}^{k+1}\\ =\,&\textrm{P}_{{\mathbb {R}}^m_+}\ \left\{ {\hat{\textbf{b}}}_t+\textrm{sign}({\textbf{q}})\left[ \vert {\textbf{q}}\vert - \frac{\lambda }{\tau +\eta +\frac{1}{\alpha }} \right] _+\right\} \end{aligned} \end{aligned}$$

$$\square$$

**Algorithm 1:**
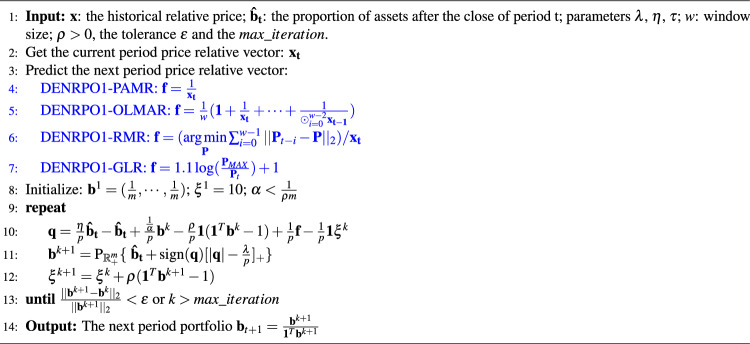
LALM for DENRPO model (13).

The above iterative update process is summarized into Algorithm 1. We point out that our proposed algorithm does not need to compute the projection onto the simplex set. This enables the computationally efficiency of our proposed algorithm.

It can be proved that the LALM can be equivalently reformulated as the Chambolle-pock algorithm^43^. Thus, we can easily get the following theorem regarding the convergence of the proposed algorithm.

##### Theorem 2

Let $\{{\textbf{b}}^k:k\in {\mathbb {N}}\}$ be generated by Algorithm 1. Then, there exists a ${\textbf{b}}^*\in {\mathbb {R}}^m$ such that

$$\begin{aligned}{\lim _{k\rightarrow \infty }}{\textbf{b}}^k={\textbf{b}}^* \end{aligned}$$

and ${\textbf{b}}^*$ is an optimal solution of problem (13).

In Algorithm 1, Theorem 2 can be established when parameters $\rho$ and $\alpha$ satisfy $\alpha < \frac{1}{\rho m}$ and $\rho >0$, where *m* is the number of assets. Theorem 2 indicates that our portfolio update algorithm outputs an optimal solution of model (13), which can be supported by theory.

#### Alternating direction method of multipliers (ADMM)

From the above discussion, the augmented Lagrangian function of problem (13) has a quadratic term $\frac{\rho }{2}\left( {\textbf{1}}^T{\textbf{b}}-1\right) ^2$, which will increase the computational difficulty, that is why we linearize the ALM. To further consider this problem, we apply ADMM to solve this problem, since ADMM introduces an auxiliary variable to guarantee that variables can be updated alternately, while remains applying gradient ascent to update the Lagrangian multiplier, which cleverly avoids the tedious process of solving the quadratic term. Specifically, we introduce an auxiliary variable ${\textbf{d}}\in {\mathbb {R}}^m$ to approach ${\textbf{b}}$, and decompose the iterative update problem of ${\textbf{b}}$ into a complete quadratic minimum solving problem for ${\textbf{b}}$ and a soft-threshold solving problem for ${\textbf{d}}$. We will illustrate the process in the following text.

Besides, since applying ADMM does not require the process of linearization, which leads to a higher accuracy, that it can restrict the solution in the simplex through project ${\textbf{b}}_{t+1}$ onto the simplex to form an eligible portfolio, as instructed by Duchi et al.^44^.

In addition, not all formulations of ADMM have saddle points. Few methods take bother to figure out and prove the existence of saddle point. However, we can prove that the augmented Lagrangian function based on the proposed model (13) has a saddle point, which makes the iterative formulae of ADMM appropriate. Next we reveal the approach of ADMM applied in the model (13).

We first formulate the problem (13) as

$$\begin{aligned} \begin{aligned} {\textbf{b}}^{k+1} \in&\mathop {\arg \min }\limits _{{\textbf{b}}} g_1({\textbf{b}})+g_2({\textbf{d}})\\&s.t.\ \ {\textbf{b}}={\textbf{d}} \end{aligned} \end{aligned}$$

where $$g_1({\textbf{b}})=-{\textbf{f}}^T{\textbf{b}}+\frac{\tau }{2}\vert \vert {\textbf{b}}\vert \vert _2^2 +{\textbf{I}}_{\Delta _m}({\textbf{b}})$$, $$g_2({\textbf{b}})=\lambda \vert \vert {\textbf{b}}-{\hat{\textbf{b}}}_t\vert \vert _1+\frac{\eta }{2}\vert \vert {\textbf{b}}-{\hat{\textbf{b}}}_t\vert \vert _2^2$$, and

22 $$\begin{aligned} {\textbf{I}}_{\Delta _m}({\textbf{b}})= {\left\{ \begin{array}{ll} 0, \hspace{5mm}&{}{\textbf{b}} \in \Delta _m,\\ +\infty , &{} \textrm{otherwise}. \end{array}\right. } \end{aligned}$$

By this way, the alternating direction method of multipliers (ADMM) can be applied to the problem (13) and its augmented Lagrangian function is

23 $$\begin{aligned} L({\textbf{b}},{\textbf{d}},{\textbf{y}})=g_1({\textbf{b}})+g_2({\textbf{d}})+{\textbf{y}}^T({\textbf{b}}-{\textbf{d}})+{\frac{\rho }{2}\vert \vert {\textbf{b}}-{\textbf{d}}\vert \vert ^2_2} \end{aligned}$$

The ADMM generates a new iterate $({\textbf{b}}^{k+1},{\textbf{d}}^{k+1},{\textbf{y}}^{k+1})$ by

24 $$\begin{aligned} {\left\{ \begin{array}{ll} {\textbf{b}}^{k+1}\in \mathop {\arg \min }\limits _{b}{L({\textbf{b}},{\textbf{d}}^k,{\textbf{y}}^{k})}\\ {\textbf{d}}^{k+1}\in \mathop {\arg \min }\limits _{d}{L({\textbf{b}}^{k+1},{\textbf{d}},{\textbf{y}}^{k})}\\ {\textbf{y}}^{k+1}={\textbf{y}}^{k}+\rho ({{\textbf{b}}}^{k+1}-{\textbf{d}}^{k+1}) \end{array}\right. } \end{aligned}$$

The following lemma gives the closed-form solution to the above problems.

##### Lemma 2

The closed-form solutions to ${\textbf{b}}$ and ${\textbf{d}}$ in the optimization problem (24) are

$$\begin{aligned} {\textbf{b}}^{k + 1} =&\textrm{P}_{\Delta _m}\left\{ \frac{1}{\tau + \rho } \left( {\textbf{f}}-{\textbf{y}}^k+\rho {\textbf{d}}^k \right) \right\} \ ,\\ {\textbf{d}}^{k + 1}=&{\hat{\textbf{b}}}_t+\textrm{sign}({\textbf{D}})\left[ \vert {\textbf{D}}\vert - \frac{\lambda }{\eta + \rho }\right] _+\ , \end{aligned}$$

where ${\textbf{D}} = \frac{1}{\eta + \rho }\left( {\textbf{y}}^k+\rho {\textbf{b}}^{k + 1}-\rho {\hat{\textbf{b}}}_t \right)$, and $\textrm{P}_{\Delta _m}$ is the projection onto the simplex $\Delta _m$.

##### Proof

For the iteration of ${\textbf{b}}$ in the problem (24), we can find that

25 $$\begin{aligned} \begin{aligned} {\textbf{b}}^{k + 1} \in&\mathop {\arg \min }\limits _{{\textbf{b}}\in \Delta _m} -{\textbf{f}}^T{\textbf{b}}+\frac{\tau }{2}\vert \vert {\textbf{b}}\vert \vert _2^2+{\textbf{y}}{^k}^T({\textbf{b}}-{\textbf{d}}^k)+\frac{\rho }{2}\vert \vert {\textbf{b}}-{\textbf{d}}^k\vert \vert ^2_2\\ =&\mathop {\arg \min }\limits _{{\textbf{b}}\in \Delta _m}<{\textbf{b}},-{\textbf{f}}>+<{\textbf{b}},{\textbf{y}}^k>+\frac{\tau }{2}\vert \vert {\textbf{b}}\vert \vert _2^2+\frac{\rho }{2}\vert \vert {\textbf{b}}-{\textbf{d}}^k\vert \vert ^2_2\\ =&\mathop {\arg \min }\limits _{{\textbf{b}}\in \Delta _m} \frac{1}{2}(\tau +\rho )\vert \vert {\textbf{b}}-\frac{1}{\tau +\rho } \left( {\textbf{f}}-{\textbf{y}}^k+\rho {\textbf{d}}^k \right) \vert \vert ^2_2\\ =&\textrm{P}_{\Delta _m}\left\{ \frac{1}{\tau +\rho } \left( {\textbf{f}}-{\textbf{y}}^k+\rho {\textbf{d}}^k \right) \right\} \, \end{aligned} \end{aligned}$$

For the iteration of ${\textbf{d}}$ in the problem (24), we can solve it in the following:

26 $$\begin{aligned} \begin{aligned} {\textbf{d}}^{k + 1} \in&\mathop {\arg \min }\limits _{{\textbf{d}}} \lambda \vert \vert {\textbf{d}}-{\hat{\textbf{b}}}_t\vert \vert _1+\frac{\eta }{2}\vert \vert {\textbf{d}}-{\hat{\textbf{b}}}_t\vert \vert _2^2+{{\textbf{y}}^k}^T({\textbf{b}}^{k+1}-{\textbf{d}})+\frac{\rho }{2}\vert \vert {\textbf{b}}^{k+1}-{\textbf{d}}\vert \vert ^2_2\\ =&\mathop {\arg \min }\limits _{{\textbf{d}}} \lambda \vert \vert {\textbf{d}}-{\hat{\textbf{b}}}_t\vert \vert _1+<{\textbf{d}},-{\textbf{y}}^k>+\frac{\eta }{2}\vert \vert {\textbf{d}}-{\hat{\textbf{b}}}_t\vert \vert _2^2+\frac{\rho }{2}\vert \vert {\textbf{d}}-{\textbf{b}}^{k+1}\vert \vert ^2_2\\ =&\mathop {\arg \min }\limits _{{\textbf{d}}}\lambda \vert \vert {\textbf{d}}-{\hat{\textbf{b}}}_t\vert \vert _1+\frac{1}{2}(\eta +\rho )\vert \vert {\textbf{d}}-\frac{1}{\eta +\rho } \left( {\textbf{y}}^k+\eta {\hat{\textbf{b}}}_t +\rho {\textbf{b}}^{k+1}\right) \vert \vert ^2_2 \end{aligned} \end{aligned}$$

Let ${\textbf{w}}={\textbf{d}}-{\hat{\textbf{b}}}_t$, then the formula (26) is equal to

27 $$\begin{aligned} \begin{aligned} {\textbf{w}}^{k+1} \in&\mathop {\arg \min }\limits _{{\textbf{w}}}\lambda \vert \vert {\textbf{w}}\vert \vert _1+\frac{1}{2}(\eta +\rho )\vert \vert {\textbf{w}}-\frac{1}{\eta +\rho }({\textbf{y}}^k +\rho {\textbf{b}}^{k+1}-\rho {\hat{\textbf{b}}}_t)\vert \vert ^2_2\\ =&\mathop {\arg \min }\limits _{{\textbf{w}}}\frac{\lambda }{\eta +\rho }\vert \vert {\textbf{w}}\vert \vert _1+\frac{1}{2}\vert \vert {\textbf{w}}-\frac{1}{\eta +\rho }({\textbf{y}}^k +\rho {\textbf{b}}^{k+1}-\rho {\hat{\textbf{b}}}_t)\vert \vert ^2_2\\ =&\textrm{sign}({\textbf{D}})\left[ \vert {\textbf{D}}\vert - \frac{\lambda }{\eta +\rho }\right] _+\, \end{aligned} \end{aligned}$$

where ${\textbf{D}} = \frac{1}{\eta +\rho }({\textbf{y}}^k +\rho {\textbf{b}}^{k+1}-\rho {\hat{\textbf{b}}}_t)$, then we can acquire the result:

28 $$\begin{aligned} \begin{aligned} {\textbf{d}}^{k+1}=&{\hat{\textbf{b}}}_t+{\textbf{w}}^{k+1}\\ =&{\hat{\textbf{b}}}_t+\textrm{sign}({\textbf{D}})\left[ \vert {\textbf{D}}\vert - \frac{\lambda }{\eta +\rho }\right] _+ \end{aligned} \end{aligned}$$

$$\square$$

**Algorithm 2:**
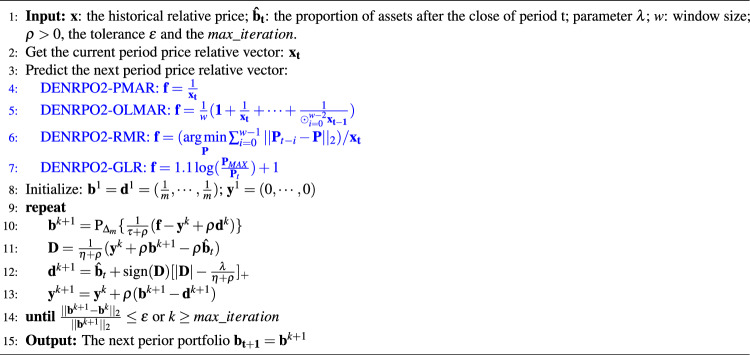
ADMM for the DENRPO model (13).

The ADMM solving problem (13) is summarized in Algorithm 2.

We next establish the convergence of ADMM applied to problem (13) for the existence of a saddle point for the Lagrangian function (23). We show this result in the following proposition.

##### Proposition 3

The Lagrangian function (23) has a saddle point, i.e., there exists $({\textbf{b}}^{*},{\textbf{d}}^{*},{\textbf{y}}^{*})$ such that

$$\begin{aligned} L({\textbf{b}}^{*},{\textbf{d}}^{*},{\textbf{y}})\le L({\textbf{b}}^{*},{\textbf{d}}^{*},{\textbf{y}}^{*})\le L({\textbf{b}},{\textbf{d}},{\textbf{y}}^{*}) \end{aligned}$$

for all $${\textbf{b}},{\textbf{d}},{\textbf{y}} \in {\mathbb {R}}^{m}$$.

##### Proof

By Theorem 1, we suppose ${\textbf{b}}^{*}$ is an optimal solution of problem (13). That is

$$\begin{aligned} {\textbf{b}}^{*}\in \mathop {\arg \min }\limits _{{\textbf{b}}} g_1({\textbf{b}})+g_2({\textbf{b}}). \end{aligned}$$

Then, the Fermat’s rule leads to ${\textbf{0}} \in \partial {(g_1+g_2)({\textbf{b}}^{*})}$. Besides, it is obviously that $g_1$ and $g_2$ can be easily proved their convexity in Theorem 1. Since $g_1$ and $g_2$ are convexity and

$$\begin{aligned} dom(g_2):=\{ {\textbf{x}} \in {\mathbb {R}}^{m}:g_2(x) < +\infty \}={\mathbb {R}}^m, \end{aligned}$$

we have ${\textbf{0}} \in \partial {g_1({\textbf{b}}^{*})}+\partial {g_2({\textbf{b}}^{*})}$. Thus, there exists

$$\begin{aligned} {\textbf{y}}^* \in \partial {g_2({\textbf{b}}^{*})}\ \ \textrm{such}\ \textrm{that}\ -{\textbf{y}}^{*} \in \partial {g_1({\textbf{b}}^{*})}. \end{aligned}$$

Set ${\textbf{d}}^{*}={\textbf{b}}^{*}$. We next show $({\textbf{b}}^{*},{\textbf{d}}^{*},{\textbf{y}}^{*})$ is a saddle point of $L({\textbf{b}},{\textbf{d}},{\textbf{y}}^*)$. First, let $L({\textbf{b}},{\textbf{d}},{\textbf{y}}^*)$ take the partial derivative of ${\textbf{b}}$ at ${\textbf{b}}^*$ and take the partial derivative of ${\textbf{d}}$ at ${\textbf{d}}^*$, then we can get

$$\begin{aligned} {\left\{ \begin{array}{ll} {\textbf{0}}\in \partial {g_1({\textbf{b}}^{*})+{\textbf{y}}^{*}+\rho ({\textbf{b}}^{*}-{\textbf{d}}^{*})}, \\ {\textbf{0}}\in \partial {g_2({\textbf{d}}^{*})-{\textbf{y}}^{*}+\rho ({\textbf{d}}^{*}-{\textbf{b}}^{*})}, \end{array}\right. } \end{aligned}$$

which leads to $({\textbf{b}}^{*},{\textbf{d}}^{*})$ is a minimizer of $L({\textbf{b}},{\textbf{d}},{\textbf{y}}^{*})$ due to the convexity of $L({\textbf{b}},{\textbf{d}},{\textbf{y}}^{*})$. This implies that

29 $$\begin{aligned} L({\textbf{b}}^{*},{\textbf{d}}^{*},{\textbf{y}}^{*})\le L({\textbf{b}},{\textbf{d}},{\textbf{y}}^{*}) \end{aligned}$$

for all ${\textbf{b}},{\textbf{d}}\in {\mathbb {R}}^m$. Second, the proposed model (13) is strongly convex if the regularization parameters satisfy. By Slater’s theorem^45^, strongly duality holds and there guarantees that

30 $$\begin{aligned} L({\textbf{b}}^{*},{\textbf{d}}^{*},{\textbf{y}}^{*})\ge L({\textbf{b}}^{*},{\textbf{d}}^{*},{\textbf{y}}) \end{aligned}$$

for any ${\textbf{y}} \in {\mathbb {R}}^m$ as ${\textbf{b}}^{*}={\textbf{d}}^{*}$. Combining (29) and (30), we complete the proof. $\square$

To integrately illustrate the ADMM applied in the proposed model (13), we are now ready to establish the convergence result in the following theorem, which is a direct consequence of Proposition 3 and Proposition 5.4.1 in^46^.

##### Theorem 4

Let $\{\left( {\textbf{b}}^{k},{\textbf{d}}^{k},{\textbf{y}}^{k}\right) :k\in {\mathbb {N}}\}$ be generated by Algorithm 2. Then, $\{{\textbf{b}}^k,{\textbf{d}}^k,{\textbf{y}}^k\}$ is a convergent series, $\{{\textbf{b}}^k-{\textbf{d}}^k\}$ converges to ${\textbf{0}}$, and $\{{\textbf{b}}^k\}$ converges to an optimal solution of problem (13).

## Experiment

### Data-sets

We compare the performance of DENRPO and other strategies in four datasets, which are NYSE (O)^9^, NYSE (N)^12,21^, TSE^47^, MSCI^35^ and DJIA. These datasets collect the historical relative price information, where the element in *i* *th* row and *j* *th* column denotes the relative price of *j* *th* asset in *i* *th* period. NYSE (O) and NYSE (N) are the data collected from the New York Exchange, NYSE(O) contains 36 stocks ranging from 7 March 1962 to 31 December 1984 and NYSE(N) contains 23 survived till 30 June 2010. TSE comes from Toronto Stock Exchange and contains 88 stocks ranging from 4 January 1994 to 31 December 1998. MSCI contains 24 indices that represent the equity markets of 24 countries around the world, ranging from 1 April 2006 to 31 March 2010. The finally dataset DJIA collects the Dow Jones Industrial index of 30 stocks in the whole 2010. The first four datasets mainly test the performance of algorithms in the stock market, and the last dataset is applied for testing algorithms in the long-short transaction. These datasets are publicly available from the real market, so it is effective and comparable to evaluate the proposed optimization model in these datasets.

### Experimental setting

#### Parameter setting

In the proposed model, there are three regularized parameters of model, namely $\lambda$, $\eta$ and $\tau$, and four parameters for algorithm which are $\xi$, ${\textbf{y}}$, $\alpha$ and $\rho$. Thereinto, $\alpha$ is an inner variable, $\xi$ is alternately updated by dual ascent method in LALM and ${\textbf{y}}$ is the Lagrangian multiplier updated by ADMM applied in the proposed model. Therefore, the above variables will not affect the performance of the methods so that we do not discuss them. In order to control the iteration, we set the tolerance $\epsilon =10^{-8}$ and the $max \_ iteration=10^8$. We take $\alpha =\frac{0.999}{\rho m}$, $\rho =0.618$ and discuss $\lambda$, $\eta$ and $\tau$ in the following to control the regularization.

We apply the method that fixes two parameters and then change the other to determine the value of each parameter. Assuming that the transaction cost is denoted to $\gamma$, for the value of $\lambda$, referring to $\lambda =10\gamma$ in the TCO framework^31^, we fixed $\eta =\tau =0$ and search around $\lambda =10\gamma$. Our experiments show that cumulative wealth is relatively high in the same running time when $\lambda =10\gamma$ for which we take $\lambda =10\gamma$. For $\eta$ and $\tau$, we still fix one in 0 and change values of the other. We arrange and combine $\eta$ and $\tau$ performing better in above step and select the combination making the cumulative wealth higher in the same running time. For simplicity, Table 1 are the results of DENRPO1-OLMAR parameters debugging meanwhile Table 2 are the results of DENRPO2-OLMAR parameters debugging in $\gamma =0.5\%$, the value of the parameter result can be understood as the convergence speed of the solving algorithms. It is observing from the experimental result that $\eta =0.00025$ and $\tau =0.00005$ obtains a relatively outstanding comprehensive performance, thus we decided to apply these two values in all experiments.

#### Comparison approaches

We employ DENRPO method to solve the online portfolio selection problem based on the above four benchmark data sets. As a comparison, 12 other online portfolio selection algorithms also run in our experiment. Specifically, UBAH, BEST and BCRP are three benchmark approaches, where UBAH is able to reflect the stock price trend of real financial market. SSPO, S1, S2 and S3 are sparse strategies based on short-term investment. TCO1 and TCO2 are excellent approaches considering transaction costs, furtherly, TCO-RMR and TCO-GLR utilize RMR and GLR price prediction on the base of transaction cost optimization framework, respectively. WFDA is the portfolio strategy considering the long-short transaction, which is executed by wavelet feature engineering. The details of the algorithms and their parameter value, which are taken from the original paper or derived from numerical experiments based on the original paper, we list below:

- UBAH: Uniformly buy-and-hold strategy;
- BEST: Beststock in hindsight;
- BCRP^9^: Best Constant Rebalanced Portfolios in hindsight;
- TCO1^31^: Transaction Cost Optimization with parameters $\lambda =10\gamma$, $\eta =10$;
- TCO2^31^: Transaction Cost Optimization with parameters $\lambda =10\gamma$, $w=4$, $\eta =10$;
- TCO-RMR^31^: Transaction Cost Optimization utilizing robust L1 median reversion with parameters $\lambda =10\gamma$, $\eta =10$;
- TCO-GLR^31^: Transaction Cost Optimization utilizing GLR price prediction with parameters $\lambda =10\gamma$, $w=4$, $\eta =10$;
- SSPO^22^: Short-term Sparse Portfolio Optimization with parameters $\eta =0.005$, $\zeta =500$, $w=5$, $\lambda =0.5$, $\gamma = 0.01$;
- S1^23^: $w=5$, $\epsilon =0.001$ (NYSE(O), NYSE(N)), $\epsilon =0.01$ (TSE), $\epsilon =0.003$ (MSCI), where $\epsilon =0.003$ (MSCI) is derived from the data range of the original paper and our experimental effect and S2, S3 take the value in the same way;
- S2^23^: $w=5$, $\epsilon =0.001$ (NYSE(O), NYSE(N)), $\epsilon =0.01$ (TSE), $\epsilon =0.003$ (MSCI);
- S3^23^: $w=5$, $s_t=3$, $\epsilon =0.001$ (NYSE(O), NYSE(N)), $\epsilon =0.01$ (TSE), $\epsilon =0.003$ (MSCI).
- WFDA^6^: Risk Assessment with Wavelet Feature Engineering for High-Frequency Portfolio Trading.

### Cumulative wealth

#### Cumulative wealth with fixed transaction costs

**Figure 1:**
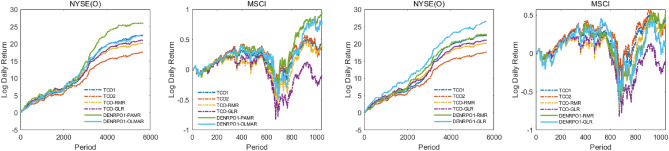
Log Daily Return obtained by DENRPO1s and TCOs with $\gamma =0.25\%$ in the NYSE(O) and MSCI.

**Figure 2:**
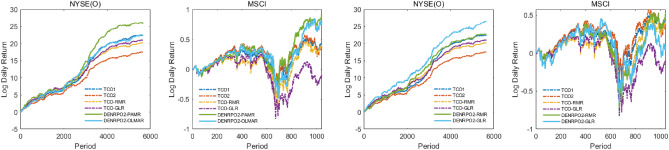
Log Daily Return obtained by DENRPO2s and TCOs with $\gamma =0.25\%$ in the NYSE(O) and MSCI.

We fix the transaction cost rate to show the daily cumulative return trend of the proposed algorithms implemented on the NYSE(O) and MSCI for simplicity, by observing the trend of wealth growth under fixed transaction costs facilitates evaluating the performance of the algorithms. Figures 1 and 2 are the daily cumulative return trend of the proposed algorithms compared with TCOs in the transaction cost rate fixed in $0.25\%$. It can be found that the wealth growth structures of DENRPO and TCO strategies are roughly the same, but the daily return of DENRPO can always be a little higher than that achieved by TCO in most cases. Thus after investing for a period, DENRPO can always obtain higher cumulative wealth, which demonstrates the superiority and practicality of the proposed method.

#### Cumulative wealth with varying transaction costs

**Figure 3:**
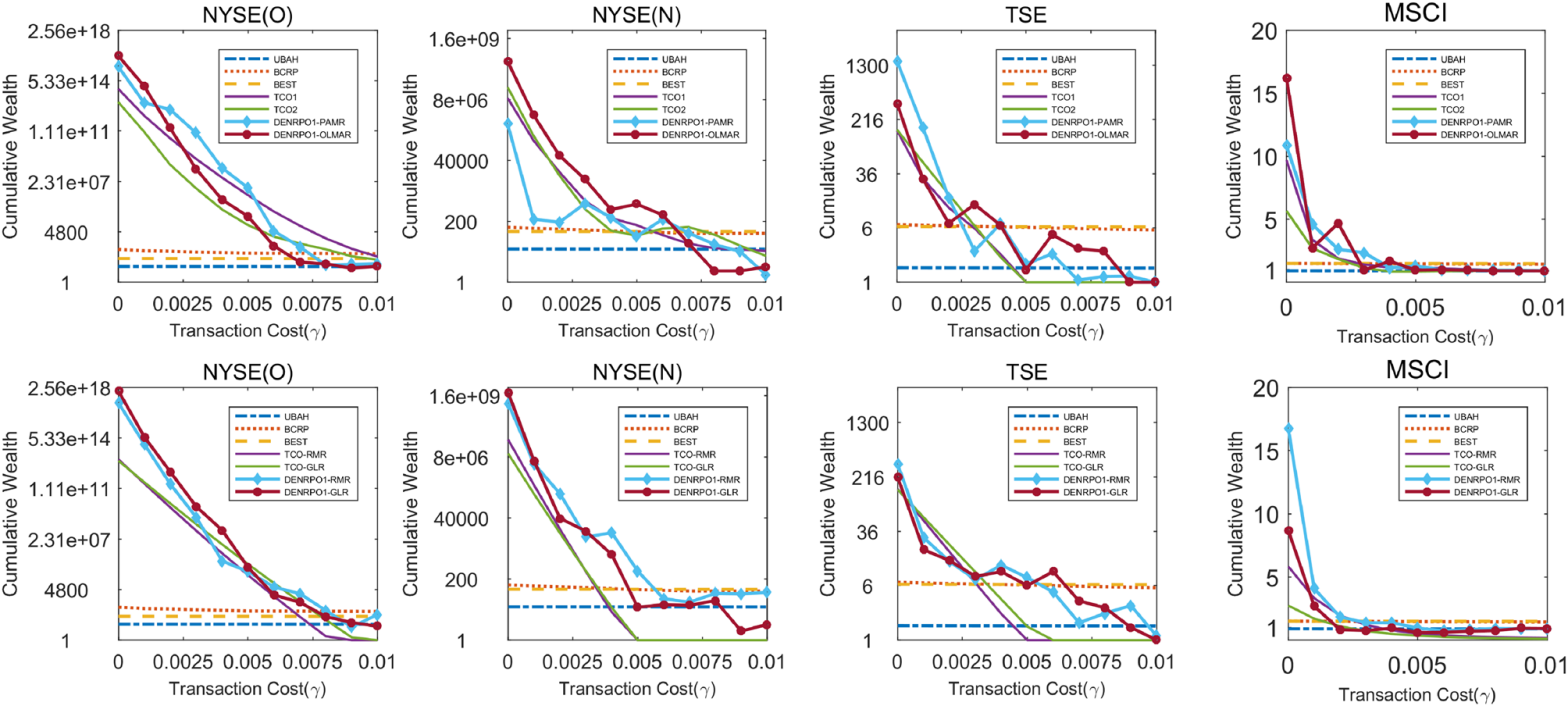
Cumulative wealth obtained by DENRPO1s and compared with cumulative wealth obtained by the listed algorithms in variable transaction costs in the four data-sets.

**Figure 4:**
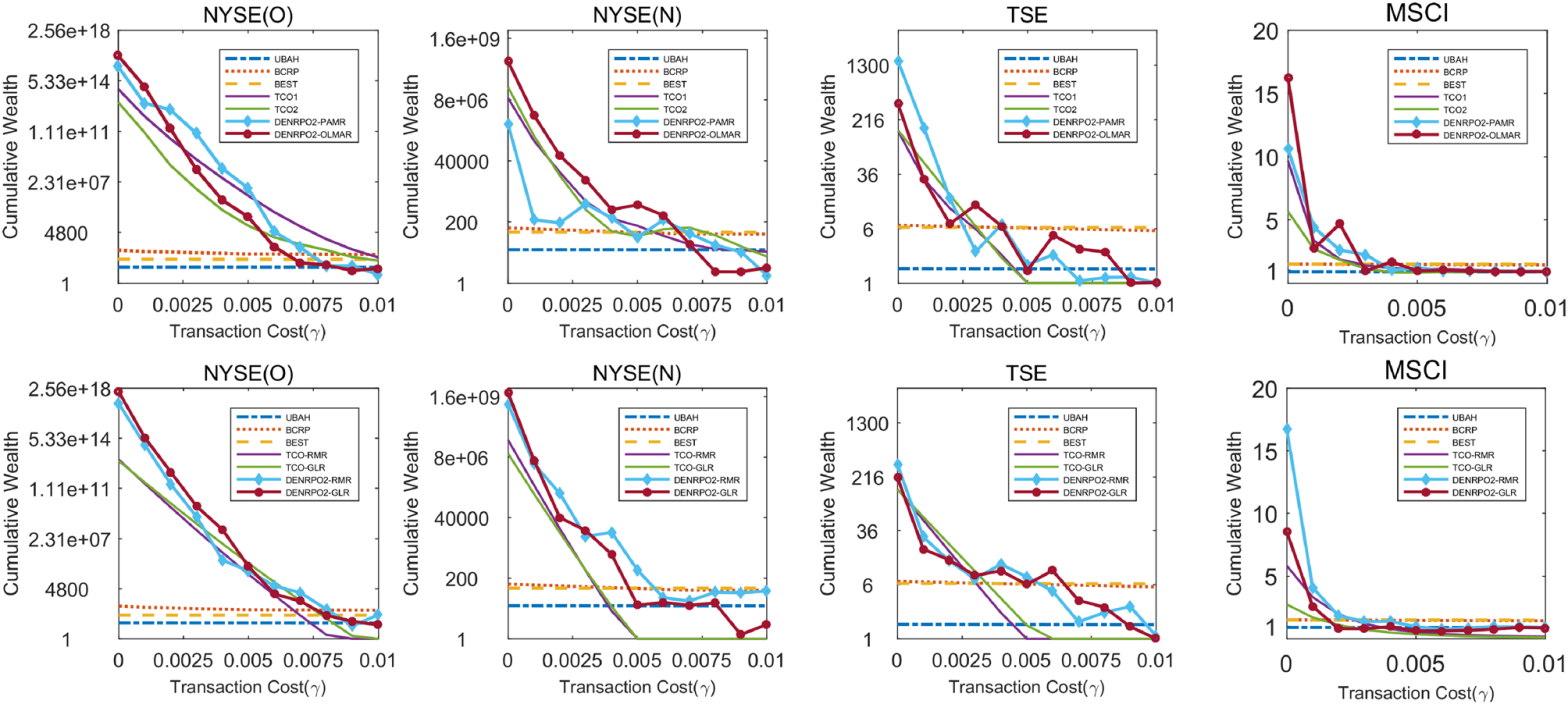
Cumulative wealth obtained by and DENRPO2s compared with cumulative wealth obtained by the listed algorithms in variable transaction costs in the four data-sets.

To better show the effectiveness of the introduced elastic net term for non-zero transaction costs and portfolio variable, meanwhile analyze the trend of the cumulative wealth in the condition of changing transaction costs, Figs. 3 and 4 as well as Table 3 compare the cumulative wealth achieved by the proposed DENRPO strategies and other methods we list above.

**Table 1:** Cumulative wealth achieved by DENRPO1-OLMAR about different permutations and combinations of $\eta$ and $\tau$ with transaction costs rates is 0.5%.

$\eta$	$\tau$: NYSE(O)						$\tau$: NYSE(N)					
	0	0.000025	0.00005	0.00025	0.0005	0.0025	0	0.000025	0.00005	0.00025	0.0005	0.0025
0	5.97E+04	5.98E+04	5.97E+04	5.81E+04	5.15E+04	4.65E+04	763.70	763.70	772.93	879.00	913.13	1139.95
0.0001	5.97E+04	5.98E+04	6.04E+04	6.07E+04	5.40E+04	4.93E+04	958.02	946.80	937.77	893.85	885.40	1134.85
0.00025	6.55E+04	6.57E+04	6.59E+04	6.22E+04	5.15E+04	5.41E+04	926.93	908.29	893.22	851.52	863.37	1125.12
0.0005	5.95E+04	5.92E+04	5.89E+04	5.56E+04	5.11E+04	6.09E+04	799.16	798.30	798.24	830.95	970.20	1101.40
0.001	4.91E+04	4.94E+04	4.96E+04	5.37E+04	5.57E+04	6.47E+04	1084.07	1083.87	1084.05	1091.84	1125.76	1025.02
0.0025	7.33E+04	7.32E+04	7.32E+04	7.21E+04	6.95E+04	5.45E+04	850.13	849.59	849.03	857.17	877.98	944.05

$\eta$	$\tau$: TSE						$\tau$: MSCI					
	0	0.000025	0.00005	0.00025	0.0005	0.0025	0	0.000025	0.00005	0.00025	0.0005	0.0025
0	1.47	1.47	1.47	1.48	1.51	2.25	0.90	0.90	0.90	0.90	0.93	0.97
0.0001	1.50	1.50	1.50	1.50	1.55	2.20	0.90	0.90	0.90	0.93	0.95	0.97
0.00025	1.53	1.53	1.53	1.56	1.63	2.12	0.96	0.96	0.96	0.97	0.97	0.98
0.0005	1.65	1.67	1.69	1.76	1.76	1.99	1.01	1.01	1.01	1.00	0.99	0.98
0.001	1.72	1.72	1.71	1.67	1.65	1.82	1.03	1.03	1.03	1.02	1.01	0.99
0.0025	1.27	1.27	1.27	1.28	1.29	1.51	1.05	1.05	1.05	1.04	1.04	1.01

**Table 2:** Cumulative wealth achieved by DENRPO2-OLMAR about different permutations and combinations of $\eta$ and $\tau$ with transaction costs rates is 0.5%.

$\eta$	$\tau$: NYSE(O)						$\tau$: NYSE(N)					
	0	0.000025	0.00005	0.00025	0.0005	0.0025	0	0.000025	0.00005	0.00025	0.0005	0.0025
0	5.98E+04	6.00E+04	5.99E+04	5.84E+04	5.13E+04	4.61E+04	758.25	758.25	772.41	872.58	904.87	1130.91
0.0001	5.98E+04	6.00E+04	6.08E+04	6.08E+04	5.44E+04	4.97E+04	951.31	940.15	932.08	888.76	879.25	1132.67
0.00025	6.56E+04	6.59E+04	6.61E+04	6.24E+04	5.13E+04	5.37E+04	920.54	902.00	886.46	843.87	857.22	1118.13
0.0005	5.97E+04	5.91E+04	5.91E+04	5.56E+04	5.14E+04	6.22E+04	791.93	790.25	791.95	807.45	963.15	1099.58
0.001	4.91E+04	4.92E+04	4.95E+04	5.38E+04	5.57E+04	6.53E+04	1075.81	1075.41	1075.38	1078.95	1118.15	1014.98
0.0025	7.46E+04	7.47E+04	7.46E+04	7.40E+04	7.07E+04	5.66E+04	840.15	839.16	837.41	825.62	871.43	936.81

$\eta$	$\tau$: TSE						$\tau$: MSCI					
	0	0.000025	0.00005	0.00025	0.0005	0.0025	0	0.000025	0.00005	0.00025	0.0005	0.0025
0	1.46	1.46	1.46	1.48	1.51	2.25	0.90	0.90	0.90	0.90	0.93	0.97
0.0001	1.49	1.50	1.50	1.51	1.55	2.21	0.90	0.90	0.90	0.93	0.95	0.97
0.00025	1.53	1.52	1.52	1.56	1.63	2.15	0.96	0.96	0.96	0.97	0.97	0.98
0.0005	1.65	1.67	1.69	1.76	1.76	1.99	1.01	1.01	1.01	1.00	0.99	0.98
0.001	1.72	1.72	1.71	1.67	1.64	1.82	1.03	1.03	1.03	1.02	1.01	0.99
0.0025	1.27	1.27	1.28	1.29	1.29	1.51	1.05	1.05	1.05	1.04	1.04	1.01

We can draw several observations in them. Firstly in Figs. 3 and 4, the cumulative wealth obtained by the three benchmark algorithms almost distributes in a straight line as transaction costs increase, indicating that the cumulative wealth obtained by these benchmark algorithms is less affected by transaction costs. Since UBAH and BEST will not rebalance the portfolio in the transaction period so that they will not produce the rebalancing cost, while BCRP fixes daily rebalancing and is less affected by transaction costs. Certainly for above reasons, they only can obtain less wealth.

**Table 3:** Cumulative wealth obtained by various algorithms on the four data-sets with transaction costs.

Algorithms	NYSE(O)			NYSE(N)			TSE			MSCI		
	0	0.0025	0.005	0	0.0025	0.005	0	0.0025	0.005	0	0.0025	0.005
UBAH	14.50	14.46	14.43	18.06	18.01	17.97	1.61	1.61	1.60	0.91	0.90	0.90
BEST	54.14	54.01	53.87	83.51	83.30	83.09	6.28	6.26	6.25	1.50	1.50	1.50
BCRP	252.07	181.03	133.52	119.71	98.02	80.23	6.70	6.38	6.12	1.51	1.49	1.48
SSPO	1.06E+18	2.45E+11	5.66E+04	1.62E+09	154.92	0.00	364.94	11.78	0.38	7.51	0.38	0.02
S1	1.24E+18	8.27E+10	5.53E+03	**2.97E+09**	93.65	0.00	227.29	6.73	0.2	10.17	0.43	0.02
S2	1.29E+18	8.12E+10	5.62E+03	2.87E+09	93.95	0.00	249.08	6.18	0.15	10.35	0.39	0.01
S3	1.24E+18	8.26E+10	5.52E+03	**2.97E+09**	93.46	0.00	227.66	6.76	0.20	10.16	0.42	0.02
TCO1	1.35E+14	5.57E+09	2.33E+06	9.15E+06	3.81E+03	143.47	149	7.66	0.91	9.68	1.52	1.13
TCO2	1.47E+13	4.34E+07	1.52E+04	2.35E+07	2.14E+03	57.61	152.98	**31**.**71**	4.99	5.66	1.42	0.84
TCO-RMR	1.44E+13	6.40E+08	5.40E+04	3.56E+07	2.37E+03	0.40	144.21	10.92	0.86	5.83	1.52	0.53
TCO-GLR	1.15E+13	1.55E+09	3.45E+05	1.08E+07	2.05E+03	0.79	141.56	**14**.**71**	1.56	2.74	0.89	0.35
DENRPO1-PARM	5.78E+15	2.10E+11	**7.99E+06**	9.61E+05	296.58	55.02	**1471**.**29**	6.89	1.83	10.84	**2**.**62**	**1**.**30**
DENRPO1-OLMAR	3.57E+16	7.36E+09	6.59E+04	2.19E+08	2.92E+03	**893**.**22**	365.16	3.93	1.53	16.21	2.15	0.96
DENRPO1-RMR	1.87E+17	8.41E+09	9.66E+04	8.29E+08	**7.72E+04**	403.96	326.54	7.91	**7**.**81**	**16**.**73**	1.70	0.97
DENRPO1-GLR	**1.36E+18**	**3.91E+11**	2.00E+05	2.12E+09	1.15E+04	17.45	212.21	14.29	6.04	8.69	1.12	0.60
DENRPO2-PARM	5.78E+15	2.10E+11	**8.02E+06**	9.61E+05	297.64	54.27	**1471.12**	6.89	1.84	10.61	**2.48**	**1**.**21**
DENRPO2-OLMAR	3.57E+16	7.37E+09	6.61E+04	2.19E+08	2.91E+03	**886**.**46**	365.18	3.93	1.52	16.21	2.15	0.96
DENRPO2-RMR	1.87E+17	8.42E+09	9.65E+04	8.29E+08	**7.72E+04**	412.39	326.42	7.91	**7**.**84**	**16**.**73**	1.70	0.97
DENRPO2-GLR	**1.36E+18**	**3.91E+11**	2.00E+05	2.24E+09	1.19E+04	18.88	212.16	14.29	6.06	8.55	1.08	0.63

Top two achievements on each column excluding benchmarks are highlighted.

Moreover, SSPO, S1, S2 and S3 perform better when transaction cost is 0 in Table 3. However, obviously find that their performance greatly reduces when operating in the non-zero transaction costs. In most data-sets, the cumulative wealth close to 0 when transaction costs only close to $0.5\%$. It is because that the goal of short-term investment is to obtain high return in a short time, thus frequent transactions are required, which will produce a amount of transaction costs. Hence, transaction costs have a greater impact on short-term investment, that leads to cumulative wealth decreases rapidly as transaction costs increase.

In addition, comparing TCO and DENRPO both transaction costs optimization strategies in Figs. 3 and 4, it can be found that the cumulative wealth of DENRPO is significantly higher than that of TCO in most cases. As transaction costs increase, DENRPO strategy can also achieve two small peaks of cumulative wealth in TSE when around $\gamma =0.3\%$ to $\gamma =0.7\%$, indicating that DENRPO strategy has the ability to counteract transaction costs, which ensures the stability and the better out-of-sample performance of the proposed method. Furtherly comparing TCO and DENRPO in Table 3, it is obviously that DENRPO obtains 8.02E+06, 893.22, 7.84, 1.30 in NYSE(O), NYSE(N), TSE and MSCI in $\gamma =0.5\%$, respectively, which indicates that DENRPO survives better than TCO in high transaction costs environment. It explains the stability of DENRPO and the significance of elastic net in the data angle.

### Mean excess return

To measure the daily return performance of each algorithm, we first need to know whether proportion of total wealth gained or lost on this day. Due to the existence of transaction costs, we develop to represent this concept as a term related to the net proportion wealth:

31 $$\begin{aligned} r_s=({\textbf{b}}_t^T{\textbf{x}}_t-1)*w_{t-1}, \end{aligned}$$

it can be understood as the net proportion gained or lost wealth. Mean excess return (MER)^48^ is defined as the average value of the daily excess returns compared with the UBAH strategy in the paper, which is given as follows:

32 $$\begin{aligned} MER={\bar{r}}_s-{\bar{r}}_m=\frac{1}{T}\sum _{t=1}^{T}r_{s,t}-r_{m,t}, \end{aligned}$$

where $r_{s,t}$ and $r_{m,t}$ are the daily excess returns of the compared portfolio strategy and the UBAH on the $t^{th}$ day, respectively.

It is obviously by the definition of MER that a superior portfolio strategy should have a larger MER value, and the larger the MER leads to the better the performance of the strategy. Certainly even a small gap in MER can indicate a larger difference in portfolio strategies, especially for the long-term investments^22^. We present the MERs for our proposed methods and the TCO strategy, which both consider the transaction cost, in Table 4.

It is obviously that DENRPO performs the best in most case, since it always gains the biggest MER. For example, the MER are 0.0069, 0.0023, 0.0060, 0.0017 in $\gamma =0.25\%$ in NYSE(O), NYSE(N), TSE and MSCI, respectively, which even many strategies that do not take into account transaction costs can not achieve. This is the reason why DENRPO outperforms other systems in cumulative wealth.

**Table 4:** Mean excess return obtained by several algorithms on the four data-sets with transaction costs.

Algorithms	NYSE(O)			NYSE(N)			TSE			MSCI		
	0	0.0025	0.005	0	0.0025	0.005	0	0.0025	0.005	0	0.0025	0.005
BEST	0.0003	0.0003	0.0003	0.0003	0.0003	0.0003	0.0016	0.0016	0.0016	0.0004	0.0004	0.0004
TCO1	0.0056	0.0051	0.0035	0.0024	0.0020	0.0013	0.0047	0.0046	0.0028	0.0024	0.0009	0.0003
TCO2	0.0052	0.0038	0.0020	0.0026	0.0017	0.0010	0.0048	0.0050	0.0034	0.0019	0.0007	0.0001
TCO-RMR	0.0053	0.0052	**0**.**0052**	0.0027	0.0027	**0**.**0026**	0.0048	0.0048	0.0048	0.0019	0.0018	**0**.**0017**
TCO-GLR	0.0054	0.0053	**0**.**0052**	0.0026	0.0026	**0**.**0025**	0.0049	0.0049	0.0049	0.0012	0.0011	**0**.**0010**
DENRPO1-PARM	0.0065	**0**.**0069**	0.0038	0.0022	0.0023	0.0014	**0**.**0068**	**0**.**0060**	0.0039	0.0026	**0**.**0017**	0.0005
DENRPO1-OLMAR	0.0069	0.0052	0.0024	0.0031	0.0021	0.0015	0.0059	0.0040	0.0027	0.0029	0.0012	0.0001
DENRPO1-RMR	0.0072	0.0053	0.0028	0.0033	**0**.**0028**	0.0016	0.0058	0.0047	0.0043	**0**.**0030**	0.0012	0.0003
DENRPO1-GLR	**0**.**0076**	0.0064	0.0033	**0**.**0035**	0.0027	0.0014	0.0056	0.0052	**0**.**0049**	0.0024	0.0008	0.0000
DENRPO2-PARM	0.0065	**0**.**0069**	0.0038	0.0022	0.0023	0.0014	**0**.**0068**	**0**.**0060**	0.0039	0.0025	**0**.**0017**	0.0004
DENRPO2-OLMAR	0.0069	0.0052	0.0024	0.0031	0.0021	0.0015	0.0059	0.0040	0.0027	0.0029	0.0012	0.0001
DENRPO2-RMR	0.0072	0.0053	0.0028	0.0033	**0**.**0028**	0.0016	0.0058	0.0047	0.0043	**0**.**0030**	0.0012	0.0003
DENRPO2-GLR	**0**.**0076**	0.0064	0.0033	**0**.**0035**	0.0027	0.0014	0.0056	0.0052	**0**.**0049**	0.0024	0.0008	0.0000

Top two achievements on each column excluding benchmark are highlighted.

### $\alpha$ Factor

We evaluate our proposed method whether outperforms the benchmark and the TCO method considering transaction costs in a statistical significance. The Capital Asset Pricing Model (CAPM)^49^ proposed that intrinsic excess return composes the part of the expected return, which is usually called $\alpha$ Factor in the finance industry^50^. $\alpha$ Factor can be improved by a excellent portfolio strategy and it can be represented in the following way:

33 $$\begin{aligned} {\hat{\beta }}=\frac{{\hat{c}}(r_s,r_m)}{{\hat{\sigma }}^2(r_s,r_m)}, \end{aligned}$$

34 $$\begin{aligned} {\hat{\alpha }}=\bar{r_s}-{\hat{\beta }}\bar{r_m}, \end{aligned}$$

where ${\hat{c}}(\cdot ,\cdot )$ and ${\hat{\sigma }}(\cdot ,\cdot )$ denote the sample covariance and the sample standard deviation (STD) computed on T trading days, respectively. Table 5 represents the $\alpha$ Factor of our proposed method compared to benchmark and TCO methods. It is obviously that DENRPO achieves 0.0067, 0.0027, 0.0057 in $\gamma =0.25\%$ in NYSE(O), NYSE(N) and TSE, respectively, which are much higher than TCO. It furtherly guarantees the better performance of the proposed methods.

**Table 5:** $\alpha$ Factor obtained by several algorithms on four data-sets with transaction costs.

Algorithms	NYSE(O)			NYSE(N)			TSE			MSCI		
	0	0.0025	0.005	0	0.0025	0.005	0	0.0025	0.005	0	0.0025	0.005
BEST	0.0003	0.0003	0.0003	0.0004	0.0004	0.0004	0.0015	0.0015	0.0015	0.0005	0.0005	0.0005
TCO1	0.0055	0.0050	0.0034	0.0023	0.0020	0.0013	0.0045	0.0044	0.0026	0.0024	0.0009	0.0003
TCO2	0.0051	0.0036	0.0019	0.0025	0.0017	0.0010	0.0046	0.0048	0.0032	0.0019	0.0007	0.0000
TCO-RMR	0.0051	0.0051	**0**.**0050**	0.0026	0.0026	**0**.**0025**	0.0045	0.0046	0.0046	0.0019	**0**.**0018**	**0**.**0017**
TCO-GLR	0.0052	0.0051	**0**.**0050**	0.0025	0.0025	**0**.**0025**	0.0047	0.0047	**0**.**0047**	0.0012	0.0011	**0**.**0010**
DENRPO1-PARM	0.0064	**0**.**0067**	0.0038	0.0021	0.0022	0.0014	**0**.**0067**	**0**.**0057**	0.0038	0.0026	**0**.**0017**	0.0005
DENRPO1-OLMAR	0.0067	0.0050	0.0024	0.0030	0.0020	0.0015	0.0057	0.0038	0.0026	0.0029	0.0012	0.0001
DENRPO1-RMR	0.0070	0.0052	0.0026	0.0032	**0**.**0027**	0.0015	0.0056	0.0044	0.0041	**0**.**0030**	0.0012	0.0002
DENRPO1-GLR	**0**.**0075**	0.0062	0.0031	**0**.**0035**	0.0026	0.0014	0.0053	0.0049	**0**.**0046**	0.0024	0.0008	0.0000
DENRPO2-PARM	0.0064	**0**.**0067**	0.0038	0.0021	0.0022	0.0014	**0**.**0067**	**0**.**0057**	0.0038	0.0025	0.0017	0.0004
DENRPO2-OLMAR	0.0067	0.0050	0.0024	0.0030	0.0020	0.0015	0.0057	0.0038	0.0026	0.0029	0.0012	0.0001
DENRPO2-RMR	0.0070	0.0052	0.0026	0.0032	**0**.**0027**	0.0015	0.0056	0.0044	0.0041	**0**.**0030**	0.0012	0.0002
DENRPO2-GLR	**0**.**0075**	0.0062	0.0031	**0**.**0035**	0.0026	0.0014	0.0053	0.0049	0.0046	0.0024	0.0008	0.0000

Top two achievements on each column excluding benchmark are highlighted.

### $\beta$ Factor

**Table 6:** $\beta$ Factor obtained by several algorithms on four data-sets with transaction costs.

Algorithms	NYSE(O)			NYSE(N)			TSE			MSCI		
	0	0.0025	0.005	0	0.0025	0.005	0	0.0025	0.005	0	0.0025	0.005
BEST	0.9169	0.9169	0.9169	0.8904	0.8904	0.8904	1.4552	1.4551	1.4551	0.4585	0.4585	0.4585
TCO1	**1**.**2092**	**1**.**2428**	1.1515	**1**.**0799**	**1**.**0398**	0.9972	1.4721	**1**.**5109**	1.5131	**1**.**1279**	**1**.**0780**	**0**.**9270**
TCO2	1.2781	1.3211	1.1964	1.1450	1.0935	1.0614	1.5308	1.5476	1.5499	1.1532	1.1601	1.1264
TCO-RMR	1.2932	1.2952	1.2968	1.1472	1.1424	1.1372	1.5563	1.5529	1.5505	1.1703	1.1686	1.1675
TCO-GLR	1.3623	1.3628	1.3643	1.1368	1.1340	1.1303	1.5832	1.5814	1.5792	1.2630	1.2654	1.2680
DENRPO1-PARM	1.2354	1.2474	**1**.**0851**	1.0855	1.0492	**0**.**9956**	**1**.**3099**	**1**.**5395**	**1**.**2146**	1.1289	**1**.**1202**	**0**.**9684**
DENRPO1-OLMAR	1.3124	1.3454	1.1309	1.1817	1.1848	1.0607	1.5291	1.5904	1.4146	1.1789	1.1297	1.0289
DENRPO1-RMR	1.2950	1.3211	1.2473	1.1541	1.1318	1.1598	1.5847	1.6075	1.5542	1.1924	1.1747	1.1089
DENRPO1-GLR	1.3462	1.3674	1.3916	1.1199	1.1329	1.1248	1.6837	1.6865	1.6147	1.2704	1.2624	1.2953
DENRPO2-PARM	**1**.**2354**	**1**.**2474**	**1**.**0852**	**1**.**0855**	**1**.**0492**	**0**.**9954**	**1**.**3099**	1.5395	**1**.**2146**	**1**.**1270**	1.1231	0.9750
DENRPO2-OLMAR	1.3125	1.3454	1.1310	1.1817	1.1849	1.0602	1.5291	1.5904	1.4139	1.1789	1.1297	1.0289
DENRPO2-RMR	1.2950	1.3211	1.2473	1.1541	1.1318	1.1593	1.5846	1.6074	1.5545	1.1924	1.1747	1.1089
DENRPO2-GLR	1.3462	1.3674	1.3916	1.1200	1.1331	1.1234	1.6837	1.6865	1.6151	1.2706	1.2629	1.2812

Top two achievements on each column excluding benchmark are highlighted.

In addition to measuring returns, we also need to measure risk indices in order to evaluate a strategy more comprehensively. $\beta$ Factor is a commonly used risk indicator, which measures the volatility of the portfolio strategy return with respect to the market benchmark. In the case of $\beta >0$ and $\beta <1$, shows that the strategy return has a positive correlation with the market return, and if $\beta$ is smaller, then the strategy return fluctuates less greatly than the market return. The calculation method of $\beta$ Factor is given in (33) and Table 6 shows the result of the proposed method compared with benchmark and TCO methods. It is obviously that DENRPO obtains 1.0851, 0.9954 and 1.2146 in $\gamma =0.5\%$ in NYSE(O), NYSE(N) and TSE, respectively, which are much smaller than TCO, indicating that the proposed method can keep the stability as transaction costs increase. This test guarantees the generalization ability of DENRPO.

### Sharpe ratio

In order to more synthetically illustrate the empirical superiority of DENRPO, we compare it with WFDA strategy in Sharpe ratio in the long-short baseline. Sharpe ratio measures the excess return in the unit risk, thus the higher ratio leads to better performance of the strategy. It can be computed as:

35 $$\begin{aligned} Sharpe\ ratio = \frac{\bar{r_s}-\bar{r_m}}{{\hat{\sigma }}^2(r_s)}. \end{aligned}$$

Besides, we compare the long-short baseline setting that simultaneously buys the top five and shorts the bottom five stocks sorted by VaR and CVaR in an hour, and holds the position for one day in DJIA dataset. Table 7 shows the Sharpe ratio of DENRPO, raw long-short term baseline and WFDA-based long-short transaction, where $\alpha$ denotes the confidence level, raw and WFDA denotes VaR and CVaR computed by raw data and WFDA processed data, respectively. We can find that the Sharpe ratio of DENRPO is the highest in all compared strategies, indicating its effectiveness and superiority in the real market.

**Table 7:** Sharpe Ratio obtained by DENRPO and WEDA on DJIA dataset in long-short transaction.

Algorithms confidence level	raw	WFDA	DENRPO1-PARM	DENRPO1-OLMAR	DENRPO1-RMR	DENRPO1-GLR
	($\times$ $10^{-3}$)	($\times$ $10^{-3}$)	($\times$ $10^{-3}$)	($\times$ $10^{-3}$)	($\times$ $10^{-2}$)	($\times$ $10^{-2}$)
$\alpha =5\%$	$-$2.3246	6.3195				
$\alpha =1\%$	$-$2.8108	6.1054	96.6294	98.0381	79.5606	79.5606
$\alpha =0.5\%$	$-$2.3246	6.3195				

Algorithms confidence level	raw	WFDA	DENRPO2-PARM	DENRPO2-OLMAR	DENRPO2-RMR	DENRPO2-GLR
	($\times$ $10^{-3}$)	($\times$ $10^{-3}$)	($\times$ $10^{-3}$)	($\times$ $10^{-3}$)	($\times$ $10^{-2}$)	($\times$ $10^{-2}$)
$\alpha =5\%$	$-$2.3246	6.3195				
$\alpha =1\%$	$-$2.8108	6.1054	96.6295	98.0380	**79**.**5621**	**120**.**8834**
$\alpha =0.5\%$	$-$2.3246	6.3195				

Top two achievements of strategies are highlighted.

## Conclusion

In this paper, we study the online portfolio selection problem with transaction costs via machine learning. First, we formulate the problem as a minimization problem on the simplex. By minimizing negative expected returns and applying elastic net regularization to transaction cost controlled terms and portfolio variables, a practical and robust model is constructed to achieve the goal of maximizing return while minimizing transaction costs. Since the augmented Lagrangian function based on the proposed model has a quadratic term, we develop to apply LALM and ADMM to solve the model, which subtly reduces the computational difficulty. Further, we theoretically guarantee that the sequences generated by the proposed algorithms converge to the solution of the proposed model, and we also establish the closed-form solutions of the subproblems in each iteration. Moreover, we compare with state-of-the-art portfolio algorithms on five commonly used benchmark datasets. Extensive numerical experiments demonstrate that the cumulative wealth obtained by proposed algorithms outperforms all compared algorithms as transaction costs increasing in most cases and it also outshine in long-short transaction scenario, indicating that our proposed portfolio selection algorithms are highly competitive and have practical significance.

## Data Availability

The datasets generated and analysed during the current study are available in the Github repository, https://github.com/Ting221/DENRPO.

## Accession codes

Code used for the numerical experiments can be found here: https://github.com/Ting221/DENRPO.
